# Long-Term Clinically Significant Posterior Capsular Opacification Development Pattern in Eyes Implanted with an Aspheric Monofocal Intraocular Lens with a Square Optic Edge

**DOI:** 10.1155/2021/4566436

**Published:** 2021-09-30

**Authors:** Javier Placeres Dabán, Juan Carlos Elvira, César Azrak, Lucía Rial, David P. Piñero, José I. Belda

**Affiliations:** ^1^Department of Ophthalmology, University Hospital of Torrevieja, Alicante, Spain; ^2^Department of Ophthalmology, University Hospital of Vinalopó, Elche, Spain; ^3^Department of Optics,Pharmacology and Anatomy, University of Alicante, Alicante, Spain

## Abstract

**Purpose:**

To analyse the posterior capsular opacification (PCO) development pattern in the long term in eyes implanted with a monofocal intraocular lens (IOL) with a square edge all around the optic.

**Methods:**

Longitudinal retrospective study is data analyzed from a total of 7059 eyes from 4764 patients (mean age: 75.8 years) undergoing cataract surgery with implantation of an aspheric monofocal IOL (Bi-Flex HL 677AB/677P, Medicontur, Budapest, Hungary). These data were retrospectively collected using the electronic medical record of the hospitals involved. Nd : YAG capsulotomy rates were calculated per year during a follow-up of more than 10 years. The Kaplan–Meier analysis was used to establish the transparent capsule survival rate.

**Results:**

The Nd : YAG capsulotomy rate increased from 1.1% at 1 year postoperatively to 17.2% at 5 years after surgery. No significant differences were found between eyes with and without capsulotomy in terms of age (*p* = 0.202), gender (*p* = 0.061), type of anaesthesia used (*p* = 0.128), and presence of conditions such as hard cataract (*p* = 0.111) or pseudoexfoliation (*p* = 0.137). IOL power was significantly lower in those eyes of patients requiring Nd : YAG capsulotomy during the follow-up (*p* < 0.001). Significantly more eyes implanted with the preloaded model of the IOL required capsulotomy (*p* < 0.001). Mean survival time and rate were 9.38 years and 85.9%, respectively.

**Conclusions:**

Most eyes undergoing cataract with implantation of the Bi-Flex IOL do not develop a clinically significant PCO requiring Nd : YAG capsulotomy in the long term. IOL material and design may be the main factors accounting for this finding.

## 1. Introduction

Posterior capsular opacification (PCO) is a relatively frequent complication after cataract surgery, which is the result of the proliferation and migration of residual crystalline epithelial cells from the posterior periphery of the capsular bag towards the space between the capsule and the optics of the intraocular lens (IOL) [1]. Its prevention is crucial since it induces a significant decrease in visual acuity and quality deterioration [2]. The material of the IOL is one relevant factor for the development of both anterior and posterior capsular opacification, with a trend to higher rates of PCO when using IOLs made of hydrophilic material instead of hydrophobic material [3–6]. Some experimental data previously reported suggested that that interleukin-6 (IL-6) contributes to the development of PCO by promoting the transformation of the growth factor *β*_2_ (TGF-*β*_2_) activation and extracellular matrix (ECM) synthesis through a JAK/STAT3 signalling-dependent mechanism [7].

Besides material, other factors, such as the design of the IOL, the configuration of the IOL optics, IOL power, and the positioning of the IOL into the capsular bag, have also great relevance [8–14]. A meta-analysis of the studies evaluating the impact of IOL design on PCO concluded that IOLs made of acrylic material and silicone, as well as those with sharp optic edges, were superior in terms of a minor incidence of PCO. In addition, some studies have confirmed the benefit of a square edge all around the optic to control cell migration [15, 16].

The prevention of PCO is crucial, and its solution is the creation of a hole in the posterior capsule (capsulotomy) using YAG laser. This hole in the posterior capsule promotes the migration of epithelial cells to the periphery and the transparency of the central area of the optic [17]. Despite YAG capsulotomy is a procedure easy to perform, it should be considered that it has some risks [18–20] and economic costs associated [21], being preferrable to delay it as much as possible. It should be considered that relevant complications, such as an accidental macular hole [19], retinal detachment, or cystoid macular oedema [20], have been described after YAG laser capsulotomy. Likewise, Nd : YAG laser can induce evident changes in PMMA IOL morphology and organic alterations in their chemistry that should be considered and controlled [18]. The objective of this study was to evaluate the long-term incidence of PCO requiring YAG capsulotomy in a large hospital population of eyes implanted with a monofocal IOL with a square edge all around the optic.

## 2. Methods

### 2.1. Patient Selection and Data Collection

Longitudinal retrospective study enrolled a total of 7059 eyes undergoing cataract surgery with implantation of a specific model of aspheric monofocal IOL (Bi-Flex HL 677AB/677P, Medicontur, Geneva, Switzerland) at the Department of Ophthalmology of the University Hospitals of Torrevieja and Elche-Vinalopó (Alicante, Spain). The primary objective of this retrospective analysis was to evaluate the incidence of PCO requiring YAG capsulotomy with this model of IOL. Clinical data were collected retrospectively using the electronic medical record (Florence) and with the help of the IT Department of the Hospitals of Torrevieja and Vinalopó. Specifically, this Department provided an anonymized database in Excel format of patients who met the study criteria during the period from January 2007 to October 2020. The study was conducted following the tenets of the Declaration of Helsinki and was approved by the ethics committee of the University Hospitals Torrevieja and Elche-Vinalopó (Alicante, Spain) (MEDICONTUR-1, data approval 25/09/2020).

Inclusion criteria were patients undergoing cataract surgery without intraoperative complications, including posterior capsular rupture, vitreous loss, retrobulbar hemorrhage, suprachoroidal effusion/hemorrhage, IOL drop or nucleus drop, and implanted with the monofocal IOL Bi-Flex HL. Exclusion criteria for the study were patients implanted with other different types of monofocal IOL, chronic or recurrent uveitis, diabetes with retinal changes, keratoconus, and endothelial corneal dystrophy.

### 2.2. Surgical Procedure

The same protocol for phacoemulsification cataract surgery was used in both hospitals involved in the study. The surgical procedure began with disinfection of the operative area using povidone iodine or chlorhexidine. After this, the surgical field was prepared, and the anaesthesia was applied through the topical use of drops or by peribulbar injection of anaesthetic depending on the potential level of collaboration of the patient. Once the surgical field was prepared, a 2.2 mm peripheral corneal incision was made manually with a calibrated knife. A viscoelastic substance was then introduced into the anterior chamber to maintain its volume, allowing the surgeon to manoeuvre with sufficient safety. At this moment, the capsulorhexis was performed using a manual technique followed by cataract partition and aspiration using different extraction techniques by microinfiltrated ultrasound through the phacoemulsifier. Afterwards, the capsule was cleaned of possible remains of cataract adhered by means of a specific irrigation-aspiration device. More viscoelastic product was injected again into the anterior chamber to avoid damaging the capsular bag with the introduction of the IOL. Finally, the aspheric monofocal IOL was introduced into the capsular bag using the MEDJET PIL-MA injector (Medicontur, Budapest, Hungary). The surgery was finished after cleaning the anterior chamber by means of an irrigation-aspiration cannula connected to the phacoemulsifier, eliminating all possible remains, with additional prophylactic intraocular instillation of antibiotics (cefuroxime), except in case of allergy (use of vancomycin instead), and topical ocular instillation of antibiotic and anti-inflammatory drops.

### 2.3. Intraocular Lens

The Bi-Flex HL IOL (Medicontur, Budapest, Hungary) is a single-piece aspherical lens (25% water content), with a square optic edge at 360°. It is made of a copolymer of hydrophobic and hydrophilic monomers, with 25% water content, and ultraviolet (UV) absorber. The refractive index of the IOL material is 1.46 and the Abbe number is 58. Concerning its design and geometry, this IOL is biconvex, with a total diameter of 13 mm and a diameter of 6 mm in the optic zone. The haptic angle is 0*°*, with an asymmetric design with posterior vaulting. The IOL is available in optic powers from −10.0 to −1.0 D in 1.0 D steps, from 0.00 to 30.00 D in 0.5 D steps, and from 31 to 35 D in 1.0 D increments. Two different models of this IOL were used in the current study: 677AB model, which is the conventional model, and 677P model, which is its preloaded model.

### 2.4. Statistical Analysis

Most data analysis was performed with the commercially available software package SPSS Version 22.0 (IBM Corporation, Armonk, NY, USA). The normality of data distributions was confirmed using the Kolmogorov–Smirnov test. Mean, standard deviation, and range were used to characterize the distribution of each variable evaluated in the sample. The Student *t*-test for unpaired data was used to compare quantitative variables among the groups of eyes requiring Nd : YAG capsulotomy during the follow-up and those not requiring it. The comparison of percentages for binary data (male/female, 677AB/677P, or peribulbar/topic) between groups was performed using the chi-square test. The Kaplan–Meier analysis was used to establish the transparent capsule survival time after cataract surgery and YAG capsulotomy-free interval. Statistical significance was determined using the log-rank test. This analysis was performed with the MedCalc software version 19.8 (MedCalc Software Ltd, Ostend, Belgium). All statistical tests were 2 tailed, and *p* values below 0.05 were considered statistically significant.

## 3. Results

This retrospective analysis included data from 7059 eyes from 4764 patients ranging in age from 33 to 100 years old (mean: 75.8; standard deviation, SD: 8.7 years). The distribution of the sample in terms of gender was as follows: 2413 males (50.7%) and 2351 females (49.3%). A total of 3409 (48.3%) and 3650 (51.7%) right and left eyes were included, respectively. Concerning the IOL model, a total of 2139 eyes (30.3%) and 4920 eyes (69.7%) were implanted with the 677AB and 677P models, respectively. The IOL power implanted ranged from −6.0 to 36.0 D (mean: 20.8 D; SD: 3.6 D), with a mean target postoperative refraction of −0.14 D (SD: 0.20; range: −2.00 to 0.50 D). Peribulbar anaesthesia was used in 3776 eyes (53.5%), whereas topic anaesthesia was used in the rest of the sample (3283 eyes, 46.5%). Mean follow-up for the patients included in the study was 4.5 years (SD: 1.3), ranging from 0.1 to 10.5 years.

### 3.1. YAG Capsulotomy Rate

**Figure 1:**
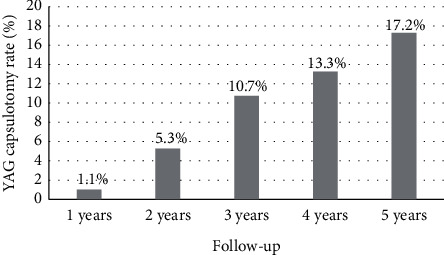
Changes in YAG capsulotomy rate during the follow-up in the sample of eyes evaluated.

Nd : YAG capsulotomy was performed in a total of 956 eyes (13.5%) of the sample retrospectively analysed.  Figure 1 shows changes in Nd : YAG capsulotomy rate during the follow-up. The YAG capsulotomy rate increased from a value of 1.1% at 1 year after the implantation of the IOL to 17.2% at 5 years postoperatively. No significant differences were found between eyes requiring Nd : YAG capsulotomy or not in terms of age (*p*=0.202), gender distribution (*p*=0.061), type of anaesthesia used (*p*=0.128), presence of hard cataract (*p*=0.111), pseudoexfoliation (*p*=0.137) or intraoperative floppy iris syndrome (IFIS) (*p*=0.382), and combined surgery of implantation of an iStent (*p*=0.352) or pars plana vitrectomy (PPV) (*p*=0.398) (Table 1). In contrast, patients requiring Nd : YAG capsulotomy were implanted with an IOL of significantly lower power (*p* < 0.001). Furthermore, significantly more eyes implanted with the 677P IOL model required Nd : YAG capsulotomy (*p* < 0.001) (Table 1).

### 3.2. Survival Analysis

**Figure 2:**
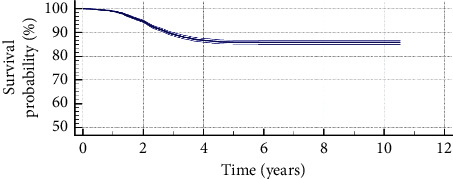
Kaplan–Meier survival curve with its confidence interval concerning transparent posterior capsule survival after cataract surgery (log-rank test: *p* < 0.01) for the sample of eyes evaluated. Mean survival time was 9.38 years for the complete follow-up, and mean survival rate was 85.9%.

**Figure 3:**
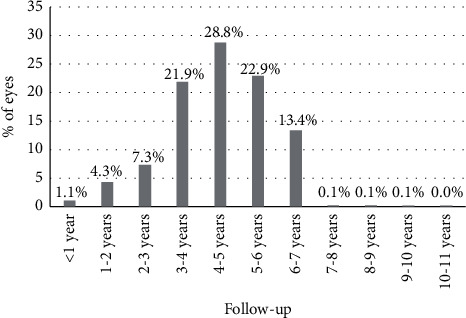
Distribution of the follow-up of cases enrolled in the current retrospective analysis.

**Figure 4:**
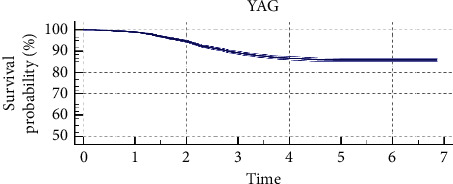
Kaplan–Meier survival curve with its confidence interval concerning transparent posterior capsule survival after cataract surgery (log-rank test: *p* < 0.01) for the sample of eyes evaluated with a follow-up of 7 years or below. Mean survival time was 6.22 years for a 7-year follow-up, and mean survival rate was 85.9%.

The Kaplan–Meier plot illustrating the transparent posterior capsule survival profile is shown in Figure 2. Most of Nd : YAG capsulotomies were performed from the first to the fourth year of follow-up after IOL implantation (843 eyes). The mean survival time was 9.38 years (standard error, 0.036; 95% confidence interval, 9.31–9.45) and the mean survival rate was 85.9% (standard error, 0.0043). As most of the patients enrolled in the study had a follow-up of 7 years or below (Figure 3), the Kaplan–Meier analysis was only repeated considering a follow-up of 7 years as maximum (Figure 4), obtaining a mean survival time of 6.22 years (standard error, 0.020; 95% confidence interval, 6.19–6.26) and a mean survival rate of 85.9% (standard error, 0.0043).

## 4. Discussion

In the current retrospective analysis, an analysis of the percentage of eyes needing Nd : YAG capsulotomy was performed in a large sample of eyes (7059 eyes) undergoing cataract surgery in a public hospital with implantation of a monofocal IOL with square optic edge at 360°. This IOL is made of a material combining hydrophobic and hydrophilic monomers. As in other retrospective studies analysing large populations [8], Nd : YAG capsulotomies were used as an estimate of clinically significant PCO. Indeed, different studies have shown similar percentage of eyes with clinically significant PCO and laser capsulotomy, with only a slight trend to obtain lower values for the Nd : YAG capsulotomy rate. Maxwell and Suryakumar [22] reported, for a hydrophobic IOL, rates of clinically significant PCO and laser capsulotomy at 3 years after surgery of 2.2% and 1.4%, respectively [4, 21, 23]. In our sample, the YAG capsulotomy rate increased from a value of 1.1% during the first year after the implantation of the IOL to 17.2% at 5 years after surgery. It should be considered that the percentage of clinically significant PCO might be slightly superior according to what was previously mentioned.

The Nd : YAG rate found in the current study was lower than that reported for different hydrophilic IOLs [3–6, 24]. Vasavada et al. [6] found Nd : YAG capsulotomy rates at 3 years after surgery of 12.9% and 16% for two different types of hydrophilic IOLs. Auffarth et al. [24] reported in a multicenter study a 3-year laser capsulotomy rate of 31.1% for a specific model of hydrophilic acrylic IOL. Furthermore, the laser capsulotomy rates of the sample evaluated were similar to those reported for some models of hydrophobic IOLs [5, 8, 25] but higher than those reported for some other models of hydrophobic acrylic IOLs [6, 10, 21, 24, 26, 27]. Hecht et al. [8] found in a large population study (14,264 cases) a Nd : YAG capsulotomy rate for a square edge hydrophobic IOL increasing from 1.1% at 1 year after surgery to 10.2% at 4 years postoperatively. Ling et al. [25] reported for another type of hydrophobic IOL a Nd : YAG capsulotomy rate increasing from 4.5% at 1 year after surgery to 12% at 3 years. However, the IOL material is not the only factor contributing to the development of PCO with a specific type of IOL. The optic edge design has been demonstrated to be a crucial factor defining the PCO development pattern [9, 10, 12, 27]. The IOL evaluated in the current series has a square optic edge at 360° that has been shown to be a continuous posterior enhanced barrier reducing the PCO rate [9].

The most significant increase in the Nd : YAG rate in our sample was found to occur during the first four years of follow-up, with a limited increase afterwards. It should be considered that the number of cases completing a follow-up of more than 7 years was reduced. This trend was consistent with the findings of other studies showing similar PCO progression rates [8, 25, 28]. Hecht and coauthors [8] showed for a specific model of hydrophobic IOL that the PCO rate increased from 1.1% at 1 year after surgery to 7.1% and 10.2% at 3 and 4 years, respectively. Praveen et al. [28] reported a significant increase of PCO rate for a hydrophobic acrylic IOL during the first 3 years after surgery, with a more limited increase afterwards.

A significant difference in IOL power was found between eyes with and without laser capsulotomy during the follow-up. Specifically, eyes requiring YAG capsulotomy were implanted with an IOL of significant lens power. In another large population study [8], significantly less PCO rates were found in eyes implanted with a hydrophobic IOL with a power of 20 D or below. Indeed, these authors identified by means of logistic regression that there was an increased risk for PCO formation with lower diopter IOLs [8]. This can be related to the anatomical dimensions of the eyes that normally need a low-power IOL which are those with long axial lengths. It should be considered that axial length has been shown to be a valuable clue to expected size of capsular bag [29], being positively correlated with capsule shrinkage [30] and capsular bag diameter [31]. Possibly, the optic edge has a more limited barrier effect in long eyes due to less stability and level of adhesion to a larger capsule with more level of shrinkage. Wang et al. [32] demonstrated that 360° anterior capsule polishing in high myopes can effectively reduce the extent of the anterior capsule contraction and increase the stability of the IOL implanted.

In the sample evaluated, no significant differences were found in age and gender between eyes requiring or not Nd : YAG capsulotomy. In addition, the type of anaesthesia used and the presence of IFIS or hard cataract were not differential factors among eyes with and without capsulotomy. Likewise, pseudoexfoliation was not related to the requirement of Nd : YAG capsulotomy in medium and long term in eyes implanted with the IOL evaluated. Østern et al. [33] also found that the development of long-term posterior capsular opacification was not increased in patients with pseudoexfoliation syndrome after uncomplicated cataract surgery. No association was found between the performance of capsulotomy in the medium and long terms and the simultaneous implantation of an iStent for the management of glaucoma or the combination with pars plana vitrectomy (PPV). Previous studies have shown that no increased PCO rate was present in eyes undergoing a combined procedure of PPV and cataract surgery, with rates even lower than those associated to eyes undergoing sequential surgeries [34]. Finally, more eyes implanted with the preloaded model of the IOL evaluated in the current sample required Nd : YAG capsulotomy to treat a clinically significant PCO. It should be considered that events such as trapped trailing haptic, problems of haptic-optic adhesion, overriding of the plunger over the optic, and trauma to optic edge have been described when using preloaded IOL implantation systems [35]. Possibly, these potential events as well as the mode of releasing the lens into the capsular bag are related to a less adjusted position of the IOL into the capsular bag. More studies are needed to corroborate if less optic edge-capsule adhesion is present in eyes implanted with the preloaded version of the IOL evaluated.

Finally, a Kaplan–Meier analysis was performed to estimate the transparent posterior capsule survival rate for the eyes implanted with the monofocal IOL evaluated. Mean survival time and rate for the whole follow-up were 9.38 years and 85.9%, respectively. Chang and Kugelberg [36] found in a comparative study that the median survival time exceeded 9 years for a hydrophobic IOL and was 2.6 years for a specific type of hydrophilic IOL. As most of the patients from the current sample had a follow-up of 7 years or less, this survival analysis was repeated considering a period of 7-year follow-up. A mean survival time of 6.22 years was obtained with this new analysis. Likewise, the survival rate was 85.9%, which was the same rate obtained considering the whole follow-up.

This study has limitations that should be acknowledged. The most important limitation is the retrospective nature of the study, limiting the type of variables that could be analysed (only those reported in the clinical histories were evaluated). Likewise, a comparative study with other types of IOLs would have been adequate to know exactly the superiority or not of the IOL evaluated in terms of PCO formation in comparison with other IOLs.

In conclusion, most of eyes undergoing cataract with implantation of the monofocal IOL evaluated in the current sample do not develop a clinically significant PCO requiring Nd : YAG capsulotomy, with a mean transparent capsule survival rate of 85.9%. The capsulotomy rate of this IOL increases over time during the four first years after surgery, with a minimal increase in the long term and a PCO rate maintained below 20%. Eyes implanted with low IOL powers using the preloaded design seem to be more predisposed to develop PCO for the specific IOL type evaluated in the current series. Future prospective comparative studies should be conducted corroborating these findings as well as comparing them with those obtained with other types of IOLs.

## Figures and Tables

**Table 1:** Differences between eyes requiring YAG capsulotomy and those not needing it in different preoperative and intraoperative variables.

Mean (SD) Range	No YAG capsulotomy (6102 eyes/3914 patients)	YAG capsulotomy (956 eyes/849 patients)	*p* value
Age (years)	75.9 (8.7) 33.0 to 100.0	75.5 (9.0) 38.0 to 98.0	0.202
IOL power (D)	20.86 (3.48) −6.00 to 36.00	20.40 (4.51) −5.00 to 36.00	<0.001
Gender (male/female) % male	1962/1952 50.1%	451/398 53.1%	0.061
Anaesthesia (peribulbar/topic) % peribulbar	3246/2854 53.2%	530/429 55.3%	0.128
Simultaneous implantation iStent (yes/no) % yes	132/5971 2.1%	23/933 2.4%	0.352
Presence of hard cataract (yes/no) % yes	357/5746 5.8%	46/910 4.8%	0.111
Presence of pseudoexfoliation (yes/no) % yes	57/6046 0.9%	5/951 0.5%	0.137
Simultaneous PPV (yes/no) % yes	61/6040 1.0%	8/950 0.8%	0.398
Presence of IFIS (yes/no) % yes	67/6036 1.1%	12/944 1.3%	0.382
IOL model (677AB/677P) % 677AB	1976/3985 33.1%	163/935 14.8%	<0.001

SD, standard deviation; IOL, intraocular lens; PPV, pars plana vitrectomy; IFIS, intraoperative floppy iris syndrome.

## Data Availability

The data used to support the findings of this study are available from the corresponding author upon request.
